# Effects of Intramuscular Vasopressin on Pharmacokinetics and Pharmacodynamics in Healthy Neonatal Piglets: A Dose–Response Study

**DOI:** 10.3390/children12101284

**Published:** 2025-09-24

**Authors:** Marwa Ramsie, Po-Yin Cheung, Raza Hyderi, Shrieya Praveen, Tze-Fun Lee, Megan O’Reilly, Georg M. Schmölzer

**Affiliations:** 1 Centre for the Studies of Asphyxia and Resuscitation, Neonatal Research Unit, Royal Alexandra Hospital, 10240 Kingsway Avenue NW, Edmonton, AB T5H 3V9, Canada; ramsie@ualberta.ca (M.R.); poyin@ualberta.ca (P.-Y.C.); rhyderi@ualberta.ca (R.H.); shrieya@ualberta.ca (S.P.); tzelee@ualberta.ca (T.-F.L.); moreilly@ualberta.ca (M.O.); 2 Department of Pediatrics, University of Alberta, Edmonton, AB T5H 3V9, Canada

**Keywords:** newborn, vasopressin, intravenous, intramuscular, pharmacokinetic, pharmacokinetic

## Abstract

Background: Neonatal resuscitation guidelines recommend the use of the vasopressor epinephrine during neonatal cardiopulmonary resuscitation (CPR); however, vasopressin may be a potential alternative. Successful neonatal CPR requires rapid vasopressor administration, but the current guideline-recommended routes can take several minutes to establish and require substantial skill and/or training. The intramuscular (IM) route provides rapid drug administration and does not require special skills, training, or equipment. Objective: We aimed to compare two doses of IM vasopressin to intravenous (IV) vasopressin in a healthy neonatal piglet model to examine the hemodynamic and pharmacokinetic effects. Methods: Fifteen neonatal piglets (*n* = 5/group; 1–3 days of age) were anesthetized, intubated via a tracheostomy, and randomized to 4 IU/kg IM vasopressin, 8 IU/kg IM vasopressin, or 0.4 IU/kg IV vasopressin. Various hemodynamic and cardiac function parameters were continuously recorded throughout the experiment. Blood was collected prior to drug administration and throughout the experiment for pharmacokinetic and pharmacodynamic analysis. Results: The 4 IU/kg IM vasopressin dose was ineffective in producing systemic changes in hemodynamics or cardiac function as it was poorly absorbed. The 8 IU/kg IM vasopressin dose had comparable results to IV vasopressin and was rapidly distributed to systemic circulation. Conclusions: The higher IM vasopressin dose of 8 IU/kg is effective in increasing systolic and diastolic blood pressure.

## 1. Introduction

At birth, 0.1% of term infants and up to 15% (2–3 million worldwide) of preterm infants will require extensive cardiopulmonary resuscitation (CPR) [1]. Despite receiving CPR, there is a high incidence of neonatal mortality. A retrospective review of newborns requiring CPR reported a 65% mortality rate (1526/2341) amongst newborns who required CPR [2]. Neonatal resuscitation guidelines recommend the use of the vasopressor epinephrine; however, its optimal route and dose remain unknown [3]. Current neonatal resuscitation guidelines recommend three routes of administration: the preferred routes of intravenous (IV) or intraosseous (IO) administration, or endotracheal tube (ETT) administration while IV/IO access is being established [4]. However, establishing IV/IO/ETT access can take several minutes, potentially reducing odds of survival. The intramuscular (IM) route of administration requires limited training/skill and allows for rapid drug administration, and the IM route is already universally recommended as the first-line treatment of anaphylaxis [5]. Ease of IM injection and shorter time to administration makes it a potential alternative route of administration during neonatal resuscitation.

Another critical knowledge gap is the effect of vasopressor drugs other than epinephrine during neonatal resuscitation [3]. Vasopressin might be an alternative during neonatal resuscitation as adult and pediatric studies and neonatal animal studies have reported improved survival with IV vasopressin compared to epinephrine [6,7,8,9,10,11]. Vasopressin induces systemic vasoconstriction through V_1_-receptor stimulation, increasing systemic vascular resistance (SVR) and blood pressure; however, vasopressin can potentially decrease myocardial contractility [12]. While vasopressin may be an alternative to epinephrine, its pharmacokinetic and pharmacodynamic effects when administered intramuscularly remain unknown, and there is no data on the optimal IM vasopressin dosage using a neonatal animal model. Therefore, we aimed to determine the hemodynamic, cardiac function, and pharmacokinetic effects of IM vasopressin as well as elucidate the optimal IM dose in healthy neonatal piglets.

## 2. Methods

**Figure 1:**
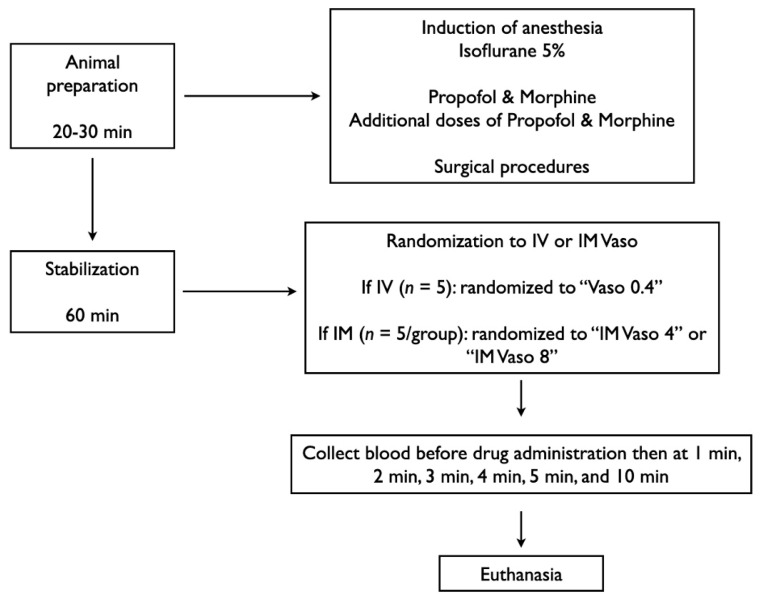
Study flow chart. IM, intramuscular; IV, intravenous; Vaso, vasopressin.

Fifteen newborn mixed breed piglets (1–3 days of age, weighing 1.7–2.4 kg) were obtained on the day of experimentation from the University Swine Research Technology Centre. All experiments were conducted in accordance with the guidelines and approval of the Animal Care and Use Committee (Health Sciences), University of Alberta [AUP00002920], presented according to the ARRIVE 2.0 guidelines, [13] and registered at preclinicaltrials.eu (PCTE0000633). A graphical display of the study protocol is presented in Figure 1.

### 2.1. Inclusion and Exclusion Criteria

Newborn mixed breed piglets 1–3 days of age with minimum weight of 1.7 kg were eligible. Piglets with congenital abnormalities or procedural complications were excluded.

### 2.2. Randomization

Piglets were randomly allocated to 0.4 IU/kg IV vasopressin, 4 IU/kg IM vasopressin, or 8 IU/kg IM vasopressin. We used these doses as their pharmacodynamic profiles via other routes were previously investigated by our group [14]. Allocation was block randomized with variable sized blocks using a computer-generated randomization program (http://www.randomizer.org). Sequentially numbered, sealed, brown envelopes containing the allocation were opened during the experiment (Figure 1).

### 2.3. Blinding

One investigator (TFL) opened the randomization envelope and was solely responsible for drug preparation. The content of the drug syringe was only known to TFL to conceal group allocation. While only TFL knew the drug allocation, we were unable to blind the different routes of administration due to the difference in administering IM and IV epinephrine. The statistical analysis was blinded to group allocation and only unblinded after the statistical analysis was completed.

### 2.4. Animal Preparation

Piglets were instrumented as previously described with modifications [15,16,17]. Following induction of anesthesia using isoflurane, piglets underwent a tracheostomy for intubation. Pressure-controlled ventilation (Sechrist Infant Ventilator Model IV-100; Sechrist Industries, Anaheim, CA, USA) was commenced at a respiratory rate of 16–20 breaths/min and pressure of 20/5 cmH_2_O. Oxygen saturation was maintained within 90–100%, while glucose was provided via an IV infusion of 5% dextrose at 10 mL/kg/hr. IV propofol (5–10 mg/kg/h) and morphine (0.1 mg/kg/h) were provided to maintain anaesthesia, and additional IV doses of propofol (1–2 mg/kg) and morphine (0.05–0.1 mg/kg) were administered as needed (i.e., to maintain adequate sedation/pain medication as determined by movement/pain response). Piglets’ normothermic body temperature of 38.5–39.5 °C was maintained with a heating pad and overhead warmer.

### 2.5. Hemodynamic Parameters

5-French Argyle^®^ (Klein-Baker Medical Inc. San Antonio, TX, USA) double-lumen and single-lumen catheters were inserted via the right femoral vein and artery, respectively. The femoral venous catheter was used for administration of fluids and medications and the arterial catheter for continuous arterial blood pressure monitoring in addition to arterial blood gas measurements. A real-time ultrasonic flow probe (2 mm; Transonic Systems Inc., Ithica, NY, USA) encircled the right common carotid artery to measure cerebral blood flow. A Millar^®^ catheter (MPVS Ultra1, ADInstruments, Houston, TX, USA) was inserted into the left ventricle via the left common carotid artery for continuous measurement of stroke volume, ejection fraction, end-diastolic and -systolic volumes, left ventricular pressure, and left ventricular contractile function (dP/dt_max_, dP/dt_min_) [18,19].

Following surgical instrumentation, piglets were placed in the supine position and allowed to recover from surgical instrumentation until baseline hemodynamic measures were stable (minimum one hour). Ventilator rate was adjusted to maintain the partial arterial CO_2_ between 35–45 mmHg, as determined by periodic arterial blood gas analysis. A Hewlett Packard 78833B monitor (Hewlett Packard Co., Palo Alto, CA, USA) was used for the continuous measurement of heart rate, mean systemic arterial pressure (MAP), systemic systolic and diastolic arterial pressure, and percutaneous oxygen saturation throughout the experiment [15,16,17].

### 2.6. Cerebral Perfusion

Cerebral oxygenation (crSO_2_) was measured using the Invos^TM^ Cerebral/Somatic Oximeter Monitor (Invos 5100, Somanetics Corp., Troy, MI, USA), which calculates crSO_2_ and expresses values as the percentage of oxygenated haemoglobin (oxygenated haemoglobin/total haemoglobin). The sensors were placed on the right forehead of the piglet and secured with wrap and tape. A slim cap provided light shielding. Regional oxygen saturation values were recorded every second at a sample rate of 0.13 Hz [17].

### 2.7. Experimental Protocol

Following surgical instrumentation and stabilization, a consecutively numbered, sealed brown envelope containing the group allocation was opened (Figure 1). Piglets were randomized into three groups: 0.4 IU/kg IV vasopressin 4 IU/kg IM vasopressin, or 8 IU/kg IM vasopressin. Piglets that were randomized to “IM” were administered one dose intramuscularly in the left inner thigh muscle while piglets randomized to “IV” were administered one dose via the femoral venous catheter followed with a 3 mL normal saline bolus. Arterial blood was collected before drug administration (baseline = time point “0 min”), 1, 2, 3, 4, 5, and 10 min after drug administration. Following final collection of blood, piglets were euthanized with an intravenous overdose of sodium pentobarbital and autopsies of IM injection sites were conducted to assess local tissue damage (120 mg/kg).

### 2.8. Data Collection and Analysis

Demographics of study piglets were recorded. Transonic flow probes, heart rate, and pressure transducer outputs, and Millar catheter were digitized and recorded with LabChart^®^ 8.1.30, programming software (ADInstruments, Houston, TX, USA). Plasma vasopressin concentrations and pharmacokinetic parameters were quantified and analyzed as previously described [14]. Reported plasma vasopressin concentrations only represent exogenous arginine vasopressin. Since pigs produce lysine vasopressin, endogenous vasopressin concentrations could not be quantified.

The data are presented as mean (standard deviation—SD) for normally distributed continuous variables and median (interquartile range—IQR) when the distribution was skewed. The data was tested for normality (Shapiro–Wilk and Kolmogorov–Smirnov test) and compared using either Student’s *t*-test (data normally distributed), Rank Sum if data were skewed, one- and two-way ANOVA with Dunnett post-test, or Fisher’s exact test for categorical variables. Both Hedges’ *g* and Cliff’s delta (δ) were reported as standardized measures of effect size as appropriate for parametric and non-parametric data, respectively. Pearson or Spearman’s correlations were reported for parametric or non-parametric data, respectively. *p*-values are 2-sided and *p* < 0.05 was considered statistically significant. Statistical analyses were performed with SigmaPlot (Systat Software Inc., San Jose, CA, USA) and R version 4.3.2.

## 3. Results

Fifteen newborn mixed breed piglets were obtained on the day of the experiment (1–3 days of age, weighing 2.0 kg (±0.2 kg)). There were no differences in the baseline parameters between the groups except for partial pressure of arterial carbon dioxide (Table 1). Autopsies post-euthanasia did not reveal any significant local tissue damage at the IM injection sites.

### 3.1. Hemodynamic Changes

**Figure 2:**
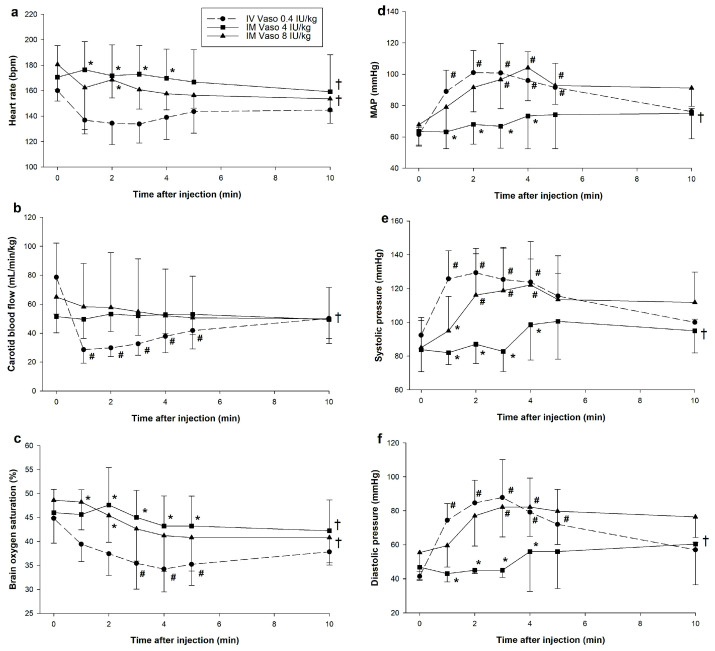
Changes in hemodynamic and blood pressure parameters following intramuscular vasopressin administration. Data are presented as mean (SD). Changes in (**a**) heart rate; (**b**) carotid blood flow; (**c**) brain oxygen saturation; (**d**) mean arterial pressure (MAP); (**e**) systolic pressure; and (**f**) diastolic pressure. # Significantly different from baseline; * Significantly different from IV vasopressin at the concurrent time point; † Significantly different from IV vasopressin over time (*p* < 0.05). IM, intramuscular; IV, intravenous; MAP, mean arterial pressure; Vaso, vasopressin. Heart rate, carotid blood flow, brain oxygen saturation, mean arterial pressure, systolic and diastolic pressure percent change from baseline following intramuscular vasopressin administration.

**Figure 3:**
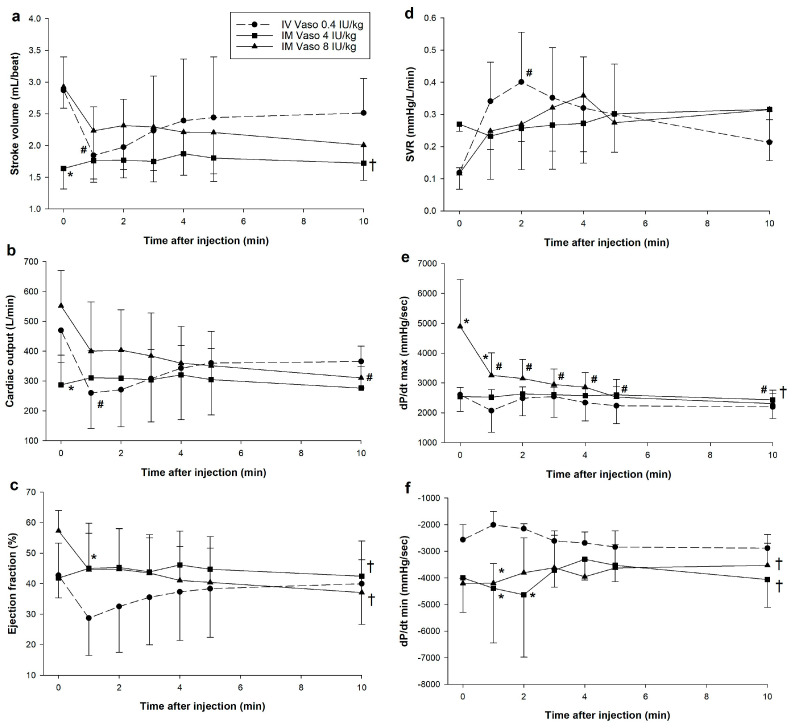
Changes in hemodynamic and cardiac function parameters following intramuscular vasopressin administration. Data are presented as mean (SD). Changes in (**a**) stroke volume; (**b**) cardiac output; (**c**) ejection fraction; (**d**) systemic vascular resistance (SVR); (**e**) dP/dt_max_; and (**f**) dP/dt_min_. # Significantly different from baseline; * Significantly different from IV vasopressin at the concurrent time point; † Significantly different from IV vasopressin over time (*p* < 0.05). dP/dt_max_, maximum rate of left ventricular pressure change; dP/dt_min_, minimum rate of left ventricular pressure change; IM, intramuscular; IV, intravenous; SVR, systemic vascular resistance; Vaso, vasopressin.

There were significant changes in carotid blood flow, brain oxygen saturation, MAP, systolic and diastolic pressure, cardiac output, and systemic vascular resistance (SVR) (Figure 2 and Figure 3). 4 IU/kg IM vasopressin failed to produce significant changes in any hemodynamic parameters (Figure 2 and Figure 3). Significant differences in hemodynamics and cardiac function parameters between 4 IU/kg IM vasopressin and IV vasopressin are therefore a result of changes following IV vasopressin administration. In contrast, 8 IU/kg IM vasopressin induced significant hemodynamic changes. However, carotid blood flow was much lower after IV vasopressin throughout the experimental period as compared with 8 IU/kg IM (*p* = 0.032). Carotid blood flow after IV vasopressin administration was negatively correlated with plasma vasopressin concentrations (Spearman’s ρ = −0.731, *p* < 0.001). Significantly lower brain oxygen saturation compared to baseline was observed with IV vasopressin but not 8 IU/kg IM vasopressin, likely due to greater reduction in carotid blood flow with IV vasopressin compared to 4 IU/kg IM and 8 IU/kg IM vasopressin (Figure 2b,c). Brain oxygen saturation was negatively correlated with IV vasopressin (Pearson’s r = −0.367, *p* = 0.03) but not 8 IU/kg IM vasopressin (Pearson’s r = −0.267, *p* = 0.164). Changes in MAP, systolic and diastolic pressure were comparable between IV and 8 IU/kg IM vasopressin (Figure 2d–f). Positive correlations between MAP and plasma vasopressin concentrations were observed with IV vasopressin (Pearson’s r = 0.739, *p* < 0.001) and 8 IU/kg IM vasopressin (Pearson’s r = 0.389, *p* = 0.045).

IV vasopressin had significantly lower stoke volume and cardiac output one minute after administration (Figure 3a–c). Significant reductions in cardiac output with 8 IU/kg IM vasopressin was observed 10 min after administration. There were no significant differences stroke volume, cardiac output, or systemic vascular resistance (SVR) between 8 IU/kg IM vasopressin and IV vasopressin (Figure 3a,b,d). There were no significant changes in dP/dt_min_ with IV or 8 IU/kg IM vasopressin, or significant correlations between dP/dt_min_ and plasma vasopressin concentrations with either IV (Pearson’s r = 0.216, *p* = 0.214) or 8 IU/kg IM vasopressin (Pearson’s r = −0.265, *p* = 0.260). Unlike IV vasopressin, dP/dt_max_ was significantly reduced following 8 IU/kg IM vasopressin administration, indicating depression of left ventricular performance (Figure 3e).

IV vasopressin had significantly lower stoke volume, cardiac output, and ejection fraction 1–3 min after administration (Figure 3a–c). Significant reductions in cardiac output and ejection fraction with 8 IU/kg IM vasopressin were observed 5 and 10 min after administration, respectively. There were no significant differences stroke volume, cardiac output, ejection fraction, or systemic vascular resistance (SVR) between 8 IU/kg IM vasopressin and IV vasopressin (Figure 3a–d,f). There were no significant changes in dP/dt_min_ with IV or 8 IU/kg IM vasopressin, or significant correlations between dP/dt_min_ and plasma vasopressin concentrations with either IV (Pearson’s r = −0.0129, *p* = 0.942) or 8 IU/kg IM vasopressin (Pearson’s r = 0.218, *p* = 0.356). Unlike IV vasopressin, dP/dt_max_ was significantly reduced following 8 IU/kg IM vasopressin administration, indicating depression of left ventricular performance (Figure 3e).

### 3.2. Changes in Plasma Vasopressin Concentrations

**Figure 4:**
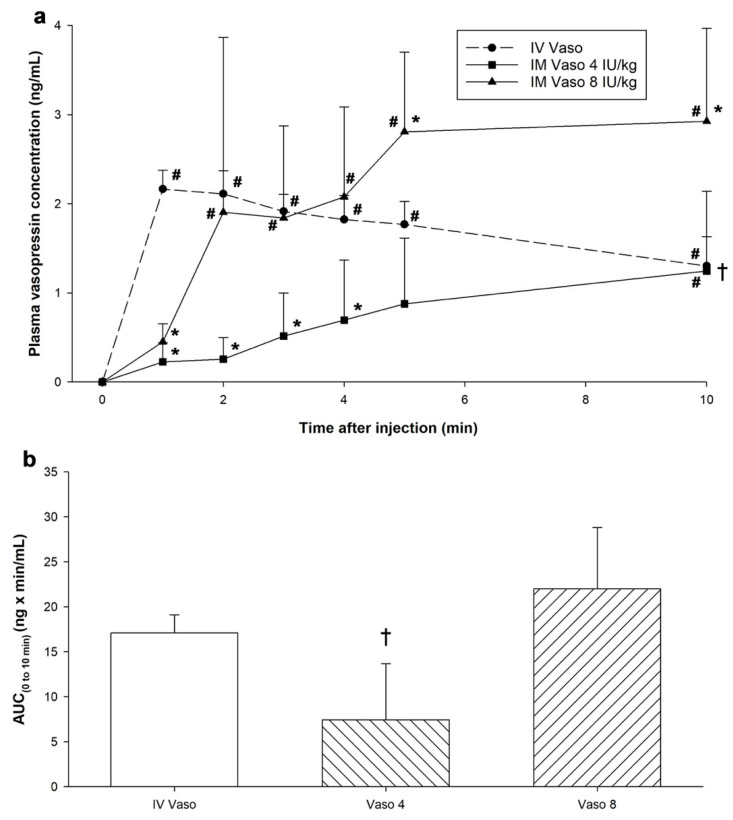
Plasma vasopressin concentrations and pharmacokinetics following intramuscular vasopressin administration. (**a**) plasma vasopressin concentration following intravenous and intramuscular administration, and (**b**) AUC (area under the curve) from 0 to 10 min (ng × minute/mL) following vasopressin administration. Data are presented as mean (SD). # Significantly different from baseline; * Significantly different from IV vasopressin at the concurrent time point; † Significantly different from IV vasopressin over time (*p* < 0.05). IM, intramuscular; IV, intravenous; Vaso, vasopressin.

Plasma vasopressin concentrations were significantly increased 1 to 10 min after IV vasopressin administration, 10 min after 4 IU/kg IM vasopressin administration, and 2–10 min after 8 IU/kg IM vasopressin administration (Figure 4a). Plasma concentrations were significantly lower with 4 IU/kg IM vasopressin than IV vasopressin (*p* < 0.001). Concentrations were lower 1 min after 8 IU/kg IM vasopressin compared to IV vasopressin (0.39 (0.30–0.66) vs. 2.20 (1.96–2.35)ng/mL, respectively, *p* < 0.001, Cliff’s δ = 1.00, 95% CI [0.74, 1.00]) but higher at 5 min (2.64 (2.06–3.718) vs. 1.74 (1.55–2.00) ng/mL, respectively, *p* = 0.043, Cliff’s δ = −0.90, 95% CI [−0.99, −0.40]) and 10 min (2.83 (1.97–3.97) vs. 1.34 (0.99–1.59)ng/mL, respectively, *p* = 0.003, Cliff’s δ = −0.90, 95% CI [−0.99, −0.40]) (Figure 4a).

### 3.3. Pharmacokinetic Parameters

Area under the curve from baseline to 10 min (AUC_0–10_), a measure of the extent of drug absorption, was significantly lower with 4 IU/kg IM vasopressin (9.29 (1.21–12.75)ng·min/mL) compared to IV vasopressin (15.93 (15.85–18.89)ng·min/mL, *p* = 0.025, Cliff’s δ = −1.00, 95% CI [−1.00, −0.82]) (Figure 4b). AUC_0–10_ was similar between 8 IU/kg IM vasopressin (20.36 (16.54–29.14) ng·min/mL) and IV vasopressin (*p* = 0.306, Hedges *g* = −1.96, 95% CI [−3.57, −0.36]). As drug elimination had not yet begun for IM vasopressin, comparisons between pharmacokinetic parameters including maximum plasma concentration, time to maximum plasma concentration, and half-life could not be determined.

## 4. Discussion

Early vasopressor administration is critical during neonatal resuscitation. In an analysis of pediatric patients with out-of-hospital non-shockable cardiac arrest, there was a 9% decrease in odds of survival (odds ratio 0.91; 95% CI 0.81–1.01) for every minute delay in epinephrine administration [20]. The IM route allows for rapid drug administration and may serve as an alternative route of vasopressor administration during neonatal resuscitation; however, whether it is a feasible route of vasopressin administration remains unknown. This paper is the first to systemically assess the dose-related pharmacokinetic and pharmacodynamic effects of IM vasopressin in a healthy neonatal piglet model.

In the current study, we determined the optimal IM vasopressin dose to be 8 IU/kg, as there were significant changes in hemodynamics and left ventricular contractile function following its administration, while there were little to no hemodynamic changes following 4 IU/kg IM vasopressin administration. Two minutes after administration of 8 IU/kg IM vasopressin plasma concentrations were comparable to IV vasopressin; however, there was continued absorption as evident by the continued increase in plasma concentrations thereafter. Even if IV access can be achieved before two minutes, IV concentrations decline faster than IM-administered vasopressin. The optimal IM vasopressin dose of 8 IU/kg decreased carotid blood flow, cardiac output, ejection fraction. These results are consistent with our previous study which examined the effects of various vasopressin doses and observed dose-dependent effects of IO and ETT vasopressin [14]. Malayan et al. examined the effects of increasing concentrations of IV vasopressin in conscious dogs and reported that only the highest dose of vasopressin (1.0 ng/kg·min, ~0.4 mIU/kg·min) was capable of producing significant pressor effects and lowering heart rate [21]. Montani et al. compared various IV vasopressin doses in healthy and baroreceptor-denervated dogs and reported dose-related decreases in heart rate and cardiac output, and increases in total peripheral resistance and MAP in healthy dogs following vasopressin administration [22]. Compared to healthy dogs, baroreceptor-denervated dogs had significantly lower decreases in heart rate (−22 (4) vs. −10 (5) beats/minute; *p* < 0.05) and cardiac output (−39.2 (3.8) vs. −22.8 (3.0) mL/kg min; *p* < 0.05), suggesting baroreflex is largely responsible for the chronotropic and cardiac output effects with vasopressin [22].

We observed significant increases in MAP, systolic and diastolic pressure following administration of 8 IU/kg but not 4 IU/kg IM vasopressin. Through its vasoconstrictor effects, vasopressin increases SVR, thereby increasing arterial blood pressure [23]. Decreased dP/dt_max_ following 8 IU/kg IM vasopressin administration suggests decreased left ventricular contractility; however, this is likely an indirect effect of vasoconstriction. In an anesthetized open-chest dog model, Nakano administered various doses of vasopressin and reported a dose-related relationship between increasing doses and reductions in contractile force; however, when coronary artery vasoconstriction was prevented, there were no changes in contractility following vasopressin administration [24]. While IM vasopressin reduced left ventricular contractile function in the current study, this may be due to the administration of vasopressin in normoxic piglets. In an isolated working rat heart model, administration of vasopressin significantly decreased dP/dt_max_ during normoxia but not hypoxia, suggesting state-dependent vasoconstrictive effects [25]. Early vasopressor administration is critical during neonatal resuscitation. In an analysis of pediatric patients with out-of-hospital non-shockable cardiac arrest, there was a 9% decrease in odds of survival (odds ratio 0.91; 95% CI 0.81–1.01) for every minute delay in epinephrine administration [20]. The IM route allows for rapid drug administration and may serve as an alternative route of vasopressor administration during neonatal resuscitation; however, whether it is a feasible route of vasopressin administration remains unknown. This paper is the first to systemically assess the dose-related pharmacokinetic and pharmacodynamic effects of IM vasopressin in a healthy neonatal piglet model.

## 5. Limitations

Piglets have several anatomical and physiological similarities to humans that make them an ideal neonatal model, [26,27] as such we expect the IM injection site and doses used in this study to produce similar effects in human neonates; however, several limitations should be considered. Our model uses piglets that were sedated/anesthetized and intubated with a tightly sealed endotracheal tube to prevent leak, which may not occur in the delivery room. As our study only examined changes that occur immediately following drug administration, potential changes that may occur hours afterwards could not be examined. Although team members were blinded to vasopressor allocation, we were unable to completely blind the intervention due to the difference in administering IM versus IV vasopressin. Baseline imbalances in PaCO_2_ may affect data interpretation. Furthermore, our study examined IM vasopressin in healthy post-transitional piglets with intact circulation and will need to be replicated in a cardiac arrest model.

## 6. Conclusions

In this dose–response study in healthy neonatal piglets, 8 IU/kg IM vasopressin had pharmacodynamic effects as 0.4 IU/kg IV vasopressin. The lower IM vasopressin dose of 4 IU/kg was ineffective in inducing systemic changes in hemodynamics and cardiac function. To the best of our knowledge, we are the first to investigate IM vasopressin in a neonatal model and determine the IM vasopressin dose of 8 IU/kg was more effective in inducing significant changes in pharmacokinetic and pharmacodynamics compared to the lower 4 IU/kg IM vasopressin dose.

## Figures and Tables

**Table 1:** Baseline characteristics.

	IV Vaso 0.4 IU/kg (*n* = 5)	IM Vaso 4 IU/kg (*n* = 5)	IM Vaso 8 IU/kg (*n* = 5)	*p*-Value
Age (days)	2 (2–3)	1 (1–2)	3 (1–3)	0.531
Weight (kg)	2.1 (1.8–2.3)	1.9 (1.7–2)	2 (1.9–2.1)	0.611
Sex (male/female)	3/2	1/4	5/0	0.066
Heart rate (bpm)	160 (152–168)	174 (148–181)	183 (162–191)	0.267
Mean arterial pressure (mmHg)	61 (58–66)	61 (56–72)	62 (60–75)	0.604
Systolic (mmHg)	94 (83–102)	80 (75–84)	84 (72–94)	0.561
Diastolic (mmHg)	41 (39–44)	48 (41–49)	55 (44–55)	0.134
Carotid blood flow (mL/kg/min)	89 (52–101)	50 (45–57)	64 (38–72)	0.310
Cerebral oxygenation (%)	46 (40–50)	44 (42–49)	49 (45–51)	0.433
pH	7.44 (7.42–7.50)	7.50 (7.49–7.56)	7.53 (7.49–7.56)	0.171
PaCO_2_ (torr)	35 (34–40)	31 (31–32)	32 (26–33)	0.017
PaO_2_ (torr)	83 (64–91)	74 (73–81)	73 (70–74)	0.608
Base excess (mmol/L)	0.7 (−0.200~4.9)	3.8 (0.1~4.5)	1.7 (1.2~3.8)	0.911
Lactate (mmol/L)	3.31 (3.00–5.11)	5.69 (4.33–6.28)	5.70 (5.38–5.79)	0.237

Data are presented as median (IQR); PaCO_2_—partial pressure of arterial carbon dioxide, PaO_2_—partial pressure of arterial oxygen.

## Data Availability

The datasets generated and analyzed for this study are available from the corresponding author (GMS), upon reasonable request.
